# Unraveling the role of non-coding rare variants in epilepsy

**DOI:** 10.1371/journal.pone.0291935

**Published:** 2023-09-27

**Authors:** Alexandre Girard, Claudia Moreau, Jacques L. Michaud, Berge Minassian, Patrick Cossette, Simon L. Girard

**Affiliations:** 1 Centre Intersectoriel en Santé Durable, University of Quebec in Chicoutimi, Saguenay, Canada; 2 CHU Sainte-Justine, Montréal, Canada; 3 Department of Neurosciences and Department of Pediatrics, University of Montreal, Montréal, Canada; 4 The Hospital for Sick Children, Department of Pediatrics, Toronto, Canada; 5 Department of Pediatrics, University of Texas Southwestern Medical School, Dallas, Texas, United States of America; 6 CHUM Research Center, Montréal, Canada; 7 Department of Neurosciences, University of Montreal, Montréal, Canada; 8 CERVO Research Center, Laval University, Québec, Canada; Chuo University, JAPAN

## Abstract

The discovery of new variants has leveled off in recent years in epilepsy studies, despite the use of very large cohorts. Consequently, most of the heritability is still unexplained. Rare non-coding variants have been largely ignored in studies on epilepsy, although non-coding single nucleotide variants can have a significant impact on gene expression. We had access to whole genome sequencing (WGS) from 247 epilepsy patients and 377 controls. To assess the functional impact of non-coding variants, ExPecto, a deep learning algorithm was used to predict expression change in brain tissues. We compared the burden of rare non-coding deleterious variants between cases and controls. Rare non-coding highly deleterious variants were significantly enriched in Genetic Generalized Epilepsy (GGE), but not in Non-Acquired Focal Epilepsy (NAFE) or all epilepsy cases when compared with controls. In this study we showed that rare non-coding deleterious variants are associated with epilepsy, specifically with GGE. Larger WGS epilepsy cohort will be needed to investigate those effects at a greater resolution. Nevertheless, we demonstrated the importance of studying non-coding regions in epilepsy, a disease where new discoveries are scarce.

## Introduction

Epilepsy is a neurological disorder characterized by epileptic seizures and spontaneous episodes of abnormal neuronal activity [1, 2]. Approximately 3% of all individuals will be affected during their lifetime [3, 4]. The vast majority of genetic epilepsies are complex traits (>98%); traits that are affected by a plethora of genomic regions. With such a large array of contributing signals, it is an arduous task to detect significant associations. Several studies used familial trios to investigate *de novo* mutations, thus leading to the association of multiple genes with the disease [5–7]. However, new variant discoveries rarely meet expectations notably in recent epilepsy studies. Only large cohorts composed of tens of thousands of individuals had some success [4, 8–13]. These studies mainly focused on common or coding variants. The overwhelming majority of studies in epilepsy use either genotyping or exome sequencing to investigate the genetic causes of the disease. Consequently, little is known concerning the implication of non-coding regions in the etiology of the disease [4, 8–13]. However, these regions were shown to have an important impact on the phenotype of an individual as they affect the expression of neighboring genes [14–18]. As a large portion of the heritability of epilepsy remains unexplained, there is a glaring need to study the impact of rare non-coding variants in epilepsy.

Since non-coding regions are so vast, a strategy to prioritize variants of interest is to investigate the impact of expression quantitative trait loci (eQTL). Studying eQTL in neurological disease is a notable challenge, mainly because eQTLs effects are tissue specific and brain tissues are mostly available post-mortem [19]. Nevertheless, progress in deep learning now allows us to predict the functional effects of variants from sequencing data without having to sample tissues from our patients [20–22]. In this study, we aimed to characterize the role of rare non-coding variants in epilepsy based on their functional effect in brain tissues using one of the most powerful deep learning algorithm, ExPecto [21]. We used whole genome sequencing (WGS) data from the Canadian Epilepsy Network (CENet) cohort to investigate deleterious rare functional variants in epileptic patients [23].

## Materials and methods

### Cohort phenotyping

The CENet cohort is composed of patients with Genetic Generalized Epilepsy (GGE) or Non-Acquired Focal Epilepsy (NAFE) collected in CHUM Research Center in Montreal and controls (unaffected Developmental Epileptic Encephalopathy (DEE) trio parents) collected in CHU Ste-Justine in Montreal and the Hospital for Sick Children in Toronto [23–26]. The patients were recruited between 2002 and 2014. Patients were diagnosed by epileptologists. The clinical epilepsy phenotype was classified according to the current classification by the International League against Epilepsy (ILAE) [27]. More specifically for NAFE, patients were at least five years of age and have experienced at least two unprovoked seizures in the six months prior to starting treatment, an MRI scan of the brain that did not demonstrate any potentially epileptogenic lesions, other than mesial temporal sclerosis. Patients with clinical and EEG characteristics meeting the 1989 ILAE syndrome definitions for GGE were included. An MRI of the brain was not required for participation. All patients were at least four years of age at the time of diagnosis. In GGE, we also included patients with Jeavons syndrome, which is an idiopathic generalized form of reflex epilepsy characterized by childhood onset, unique seizure manifestations, striking light sensitivity and possible occurrence of generalized tonic-clonic seizures. Certain cases were found with an epilepsy phenotype different from the other affected family members, hence they were marked as ‘mixed’. Only one affected GGE or NAFE patient was used per family, therefore all the individuals in this study are unrelated. We used WGS from 377 controls and 247 patients, 123 GGE, 112 NAFE and 12 mixed patients (Table 1). This study was approved by the CHUM research Center (CRCHUM) ethics committee and written informed consent was obtained for all patients (2003–1394, ND 02.058 -BSP (CA)). We did not have access to information that could allow us to identify the patients.

**Table 1:** Number of individuals for each phenotype.

Phenotype	Male	Female	Total
Controls	190	187	377
All Cases	112	135	247
GGE	52	71	123
NAFE	50	62	112
Mixed	10	2	12

### Sequencing

DNA was extracted at the time of recruitment. All samples were sequenced at the same time in 2015. Samples were sequenced for the whole genome at 30X coverage at Genome Quebec Innovation Center in Montreal. gDNA was cleaned using ZR-96 DNA Clean & ConcentratorTM-5 Kit (Zymo) prior to being quantified using the Quant-iTTM PicoGreen dsDNA Assay Kit (Life Technologies) and its integrity assessed on agarose gels. Libraries were generated using the TruSeq DNA PCR-Free Library Preparation Kit (Illumina) according to the manufacturer’s recommendations. Libraries were quantified using the Quant-iTTM PicoGreen dsDNA Assay Kit (Life Technologies) and the Kapa Illumina GA with Revised Primers-SYBR Fast Universal kit (Kapa Biosystems). The average size fragment was determined using a LabChip GX (PerkinElmer) instrument. The libraries were denatured in 0.05N NaOH and diluted to 8pM using HT1 buffer. The clustering was done on an Illumina cBot and the flowcell was run on a HiSeq 2500 for 2×125 cycles (paired-end mode) using v4 chemistry and following the manufacturer’s instructions. A phiX library was used as a control and mixed with libraries at 0.01 level. The Illumina control software used was HCS 2.2.58 and the real-time analysis program used was RTA v. 1.18.64. bcl2fastq v1.8.4 was used to demultiplex samples and generate fastq reads. The filtered reads were aligned to reference Homo_sapiens assembly b37. Each readset was aligned using BWA-MEM version 0.7.10 to create a Binary Alignment Map file (.bam). Bam files were processed to gvcf files and we performed joint calling of gvcf files that were merged into a single vcf file using GATK version 3.7–0 [28]. The vcf file was recalibrated and filtered following the GATK best practice guidelines.

### Data cleaning

Cleaning was made using plink v2.0 [29]. First, Single Nucleotide Variants (SNV) with a call rate below 98% were removed using ‘—geno 0.02’. Afterward, individuals with a genotype rate below 98% were excluded using ‘—mind 0.02’. Next, SNV that did not follow the Hardy-Weinberg equilibrium were removed using ‘—hwe 0.001’. Finally, individuals with unknown biological sex were excluded. To make the principal component analysis, further cleaning was required. Only common variants were used in the PCA (maf > 0.05) and SNV that were not in linkage disequilibrium by using ‘—indep-pairwise 50 5 0.2’. Single nucleotide variants (SNVs) were filtered based on their minor allele frequency (maf), only rare variants (maf <0.01) as assessed in our cohort as well as in gnomAD v3.1.2 (non-Finnish European) were kept [30]. Variants were liftover to GRCh38 to get the most recent frequency data with an increased non-coding DNA coverage.

### Statistical analyses

We applied ExPecto on rare variants (MAF<0.01) for three tissues related to epilepsy: hippocampus, amygdala and brain cortex (GTEx V6) for which we calculated the gene expression change median [16, 31]. ExPecto computes gene expression changes by using a neural network to predict the effect of variants on features such as transcription factors, histone marks and DNA accessibility. It then transforms those feature predictions in tissue specific gene expression changes with L2-regularized linear regression models. Gene expression changes calculated by ExPecto were used to compute a Constraint Violation Score (CVS) in accordance with the methods described in Zhou *et al*. [21]. The CVS quantifies how deleterious the gene expression change is: the higher the score, the more deleterious the variant.

The accuracy of ExPecto predictions was validated using known eQTLs from the GTEx V6p release (S1 Fig) [16]. To do so, we used two parameters, the prediction’s directionality and magnitude. Directionality is defined as whether the Single Nucleotide Variant (SNV) increases or decreases gene expression. Magnitude is defined as the absolute size of the effect in gene expression fold change (natural log). We determined that the magnitude above which the accuracy of the prediction’s directionality was perfect was 0.2 so we kept only variants with a median (for the cortex, hippocampus, and amygdala) above this threshold (S1 Fig). Analyses were replicated with the median of three non-neurological tissues: artery aorta, colon transverse and skin of body to validate the tissue specificity of the model (S2 Fig).

A binomial logistic regression was performed with the python package statsmodels v0.12.2. Sex was used as a covariate as well as a two-dimension UMAP (umap-learn v0.5.1) based on the first five principal components (plink v2.0) (S3 Fig). We had access to self-declared ethnicity from the affected individuals, which allowed us to confirm that the UMAP was accurate. Analyses were replicated by using only individuals of French-Canadian and European descent to validate that the findings were not related to population structure (S4 Fig). Those individuals were selected based on the cluster that corresponded with self-declared French-Canadians and Europeans.

## Results

**Fig 1:**
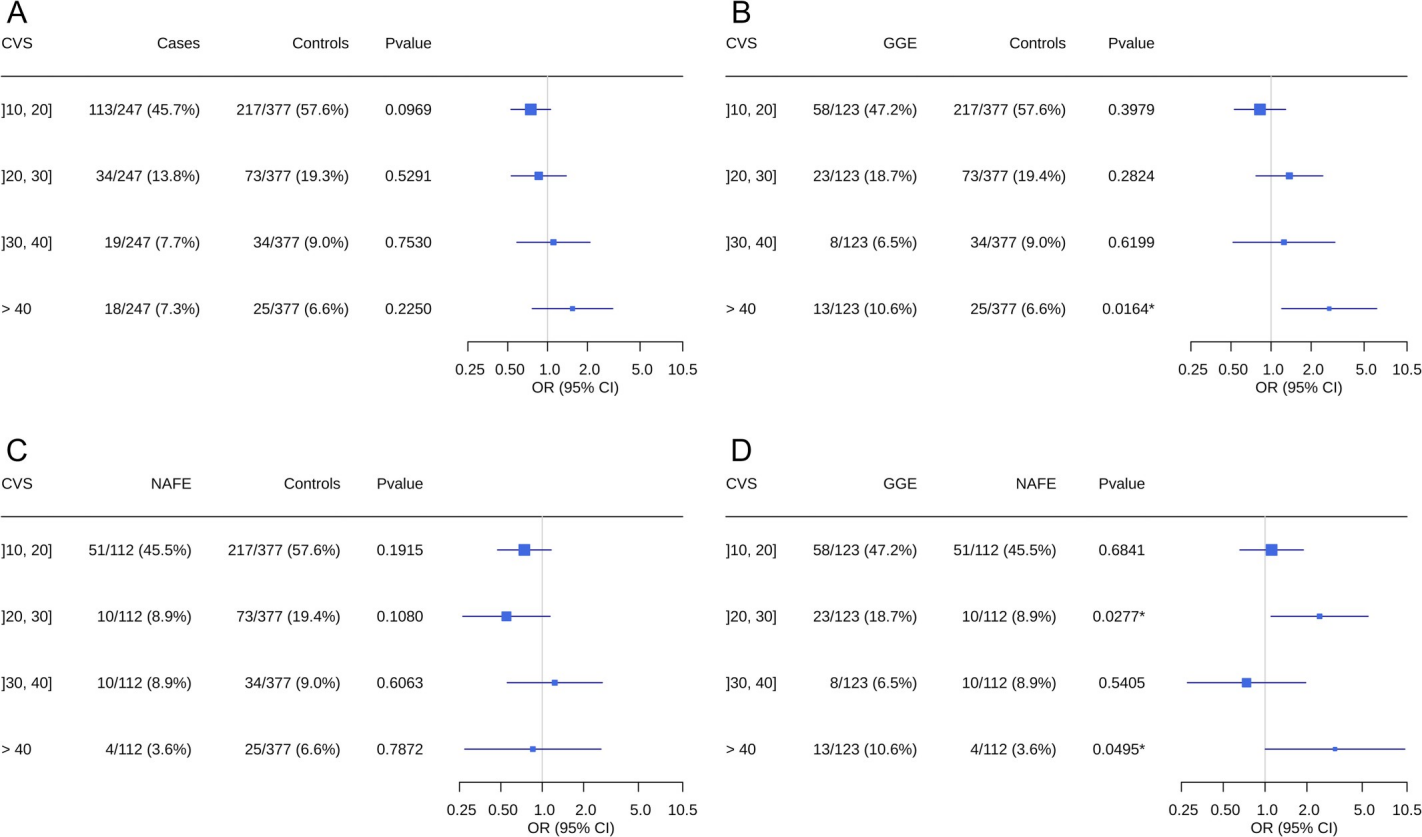
Burden of variants for different CVS thresholds across epilepsy phenotypes. Odds ratios and p-value were calculated using a binomial logistic regression for variants of different Constraint Violation Score (CVS) thresholds. Lines represent 95% confidence intervals. Comparisons were made for cases and controls (A), Genetic Generalized Epilepsy (GGE) and controls (B), Non-Acquired Focal Epilepsy (NAFE) and controls (C) and GGE and NAFE (D).

We compared the proportion of patients and controls who had at least one rare variant (MAF<0.01) at different CVS thresholds using a binomial logistic regression analysis to compute odds ratios (OR). We repeated the analysis to compare GGE with controls, NAFE with controls and GGE with NAFE (mixed patients were removed from these analyses). Variants found in the final analysis are available in S1 Table. Our analyses showed no significant difference when comparing cases and controls (Fig 1A). Nevertheless, the OR tends to increase with the CVS threshold and reaches a peak for variants with a CVS above 40 (OR 1.54; 95% CI 0.77–3.11). However, there is a significant difference between GGE and controls for CVS above 40 (OR 2.74; 95% CI 1.20–6.22) (Fig 1B). On the other hand, NAFE and controls show no significant difference, meaning that the trend observed when comparing all cases and controls was solely driven by the GGE (Fig 1C). Next, GGE and NAFE have a significantly different burden for CVS thresholds of [20, 30] (OR 2.47; 95% CI 1.10–5.52) and above 40 (OR 3.19; 95% CI 1.002–10.13) (Fig 1D). Finally, it is worth noting that none of those signals are significant after multiple tests correction, with the lowest adjusted p-value of 0.066 at CVS over 40 for GGE against controls (Bonferroni correction). Nevertheless, this particular signal is robust to multiple tests correction when using only individuals of French-Canadian and European descent with an adjusted p-value of 0.044 (S4 Fig). No significant signals were observed when repeating the analyses with non-neurological tissues, thus demonstrating the reliability and power of the tissue specific model (S2 Fig).

## Discussion

We found evidence suggesting that deleterious non-coding rare variants with a higher CVS are enriched in GGE. However, it is not the case for NAFE who are known to have a smaller genetic burden [4, 8–11]. Once again, our findings demonstrate the importance of separating GGE and NAFE when studying epilepsy, which is also supported by most associated genes being specific to subphenotypes [23, 32, 33]. Furthermore, the differences observed when directly comparing GGE to NAFE highlights the varying impact of non-coding variants on those subphenotypes.

We are among the first to highlight the potential impact of rare non-coding SNV on a genome-wide scale in epilepsy. This discovery reveals the importance of studying non-coding regions which may explain a part of the missing heritability in epilepsy [9]. Moreover, it showcases the need to conduct more studies on WGS in order to make discoveries at a higher resolution in non-coding regions.

In addition to the contribution that we bring to the field of epilepsy, the method used in this study could be applicable to other diseases to provide a better understanding of the role of rare non-coding SNV in various pathologies.

### Limitations

The study has two main limitations. First, the use of deep learning, as useful as it is, has the limitation of being predictions, not observations. Nonetheless, we validated that those predictions were accurate by using experimental data from GTEx (S1 Fig). Additionally, ExPecto is not able to compute long range (>40kb) sequence effects on gene expression, which limits this work to the study of short-range interactions. The second limitation is our small sample size. Despite this, we were successful in identifying a genome-wide effect in individuals of European descent, but we lacked power to investigate those effects at a gene or variant level resolution.

### Conclusion

This study reveals the importance of non-coding regions in the etiology of epilepsy. The effect was specific for GGE, whereas NAFE showed no significant difference with controls. Therefore, our results indicate that the differences between those subphenotypes extends to non-coding genetic mechanisms. Larger WGS cohorts will be needed to deepen our understanding of the role of non-coding regions in epilepsy.

## Supporting information

S1 Checklist STROBE statement—checklist of items that should be included in reports of observational studies. (DOCX)

S1 Fig Accuracy of predictions’ directionality on known GTEx eQTLs. Directionality accuracy was computed according to ExPecto’s predicted magnitude in natural log fold change.
(TIF)

S2 Fig Burden of variants for different CVS thresholds across epilepsy phenotypes when using non-neurological tissues. Odds ratios and p-value were calculated using a binomial logistic regression for variants of different Constraint Violation Score (CVS) thresholds. Lines represent 95% confidence intervals. Comparisons were made for cases and controls (A), Genetic Generalized Epilepsy (GGE) and controls (B), Non-Acquired Focal Epilepsy (NAFE) and controls (C) and GGE and NAFE (D). Tissues that were used are artery aorta, colon transverse and skin of body.
(TIF)

S3 Fig UMAP of ethnicity for the epilepsy patients and controls. The UMAP was made with ‘umap-learn v0.5.1’ and based on the first 5 principal components.
(TIF)

S4 Fig Burden of variants for different CVS thresholds across epilepsy phenotypes with only individuals of European descent. Odds ratios and p-value were calculated using a binomial logistic regression for variants of different Constraint Violation Score (CVS) thresholds. Lines represent 95% confidence intervals. Comparisons were made for cases and controls (A), Genetic Generalized Epilepsy (GGE) and controls (B), Non-Acquired Focal Epilepsy (NAFE) and controls (C) and GGE and NAFE (D).
(TIF)

S1 Table List of variants included in the final analysis. (XLSX)
