# Supplementary figures and images for: Unraveling the role of non-coding rare variants in epilepsy
Source: PLoS One. 2023 Sep 27;18(9):e0291935. doi: 10.1371/journal.pone.0291935 (PMC10529579; doi:10.1371/journal.pone.0291935)

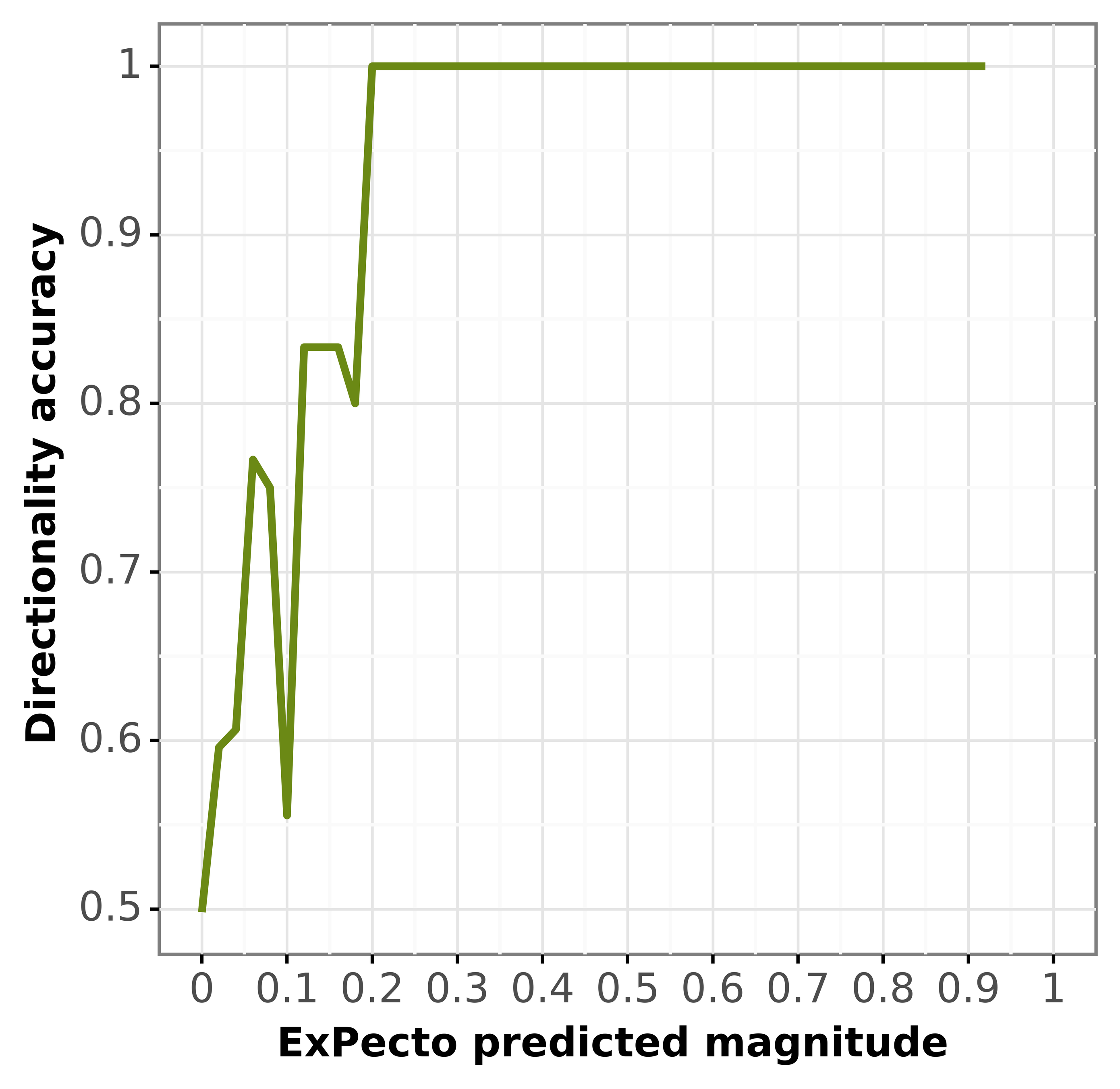

Supplement: S1 Fig — Directionality accuracy was computed according to ExPecto’s predicted magnitude in natural log fold change. (TIF) [file pone.0291935.s002.tif]

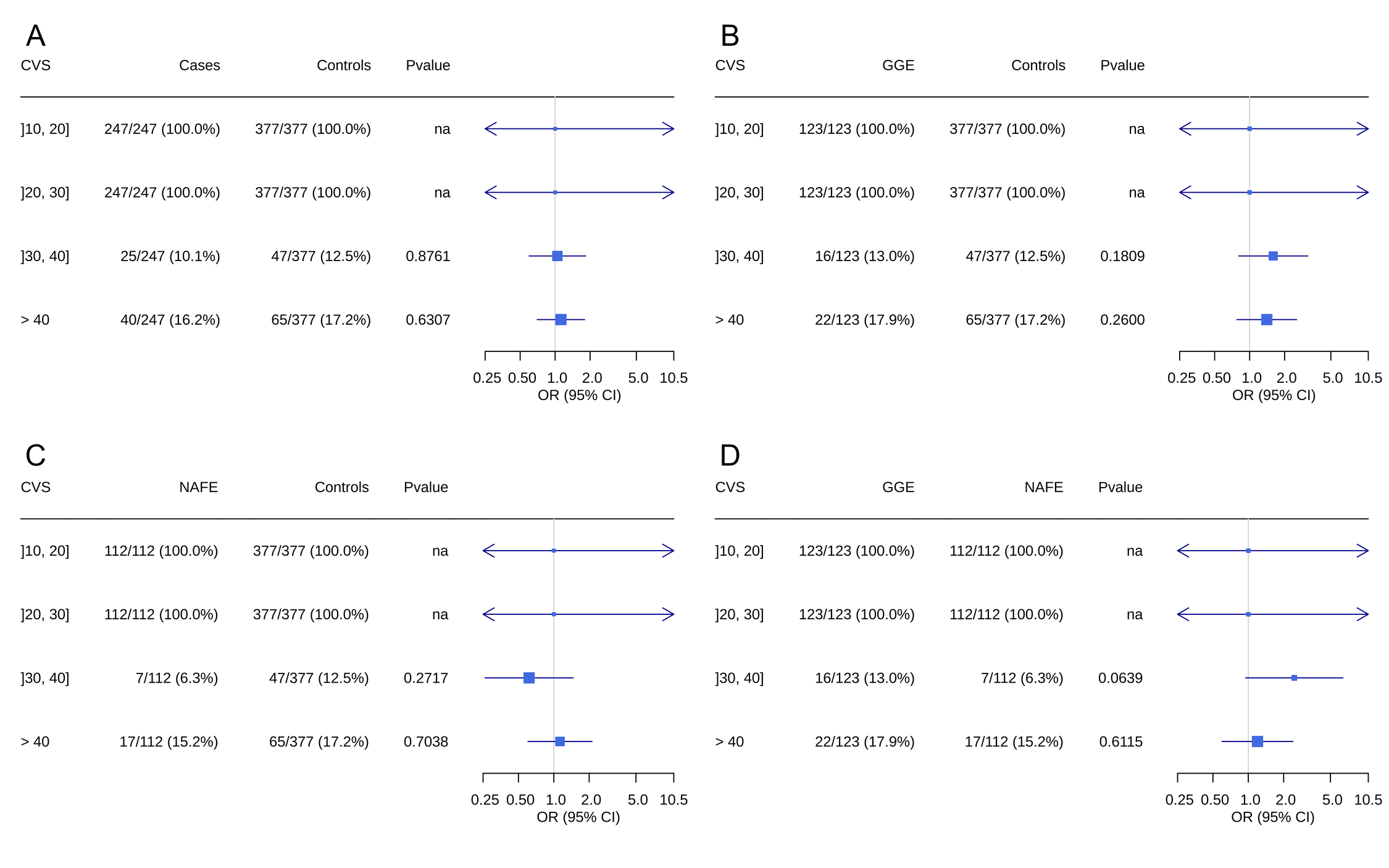

Supplement: S2 Fig — Odds ratios and p-value were calculated using a binomial logistic regression for variants of different Constraint Violation Score (CVS) thresholds. Lines represent 95% confidence intervals. Comparisons were made for cases and controls (A), Genetic Generalized Epilepsy (GGE) and controls (B), Non-Acquired Focal Epilepsy (NAFE) and controls (C) and GGE and NAFE (D). Tissues that were used are artery aorta, colon transverse and skin of body. (TIF) [file pone.0291935.s003.tif]

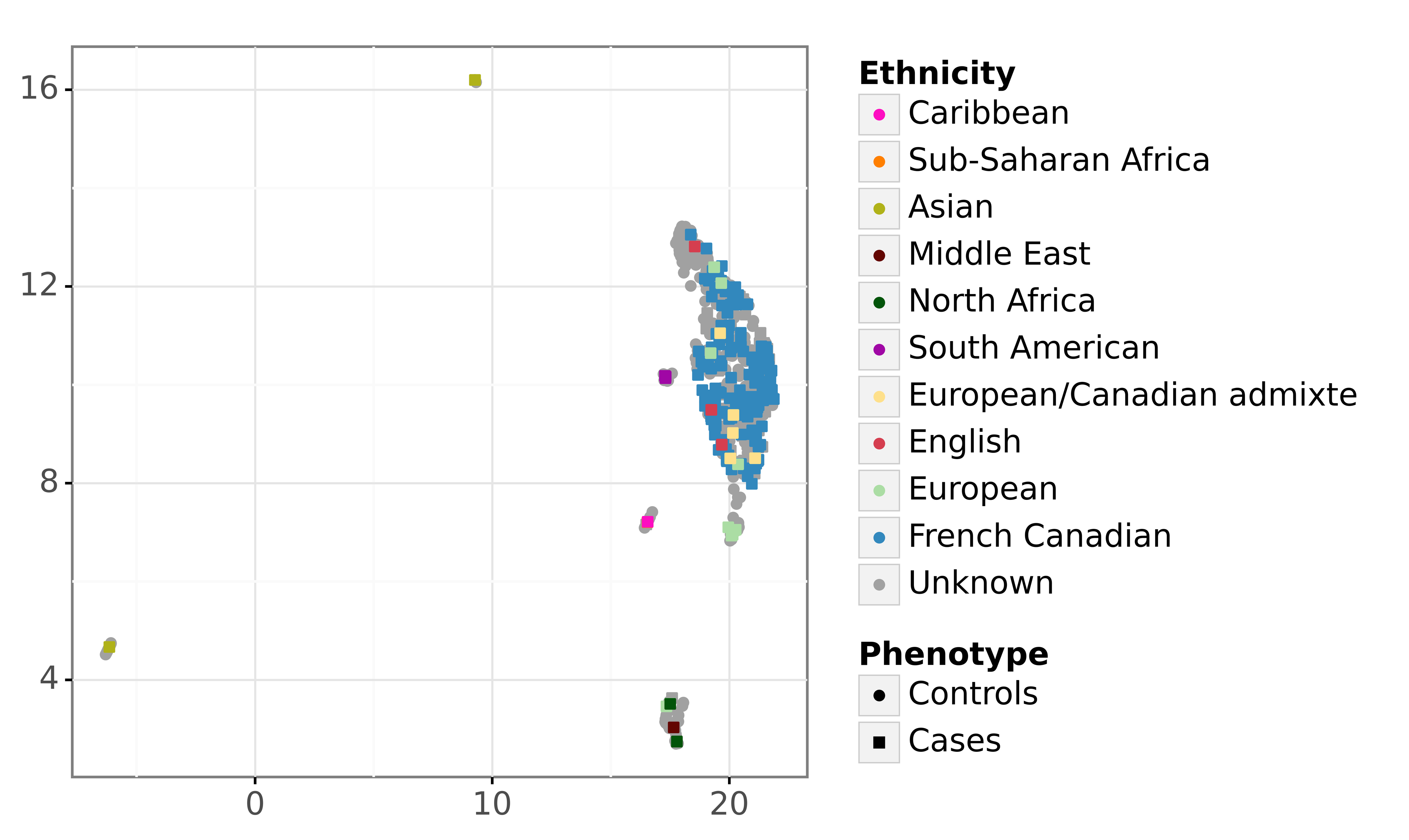

Supplement: S3 Fig — The UMAP was made with ‘umap-learn v0.5.1’ and based on the first 5 principal components. (TIF) [file pone.0291935.s004.tif]

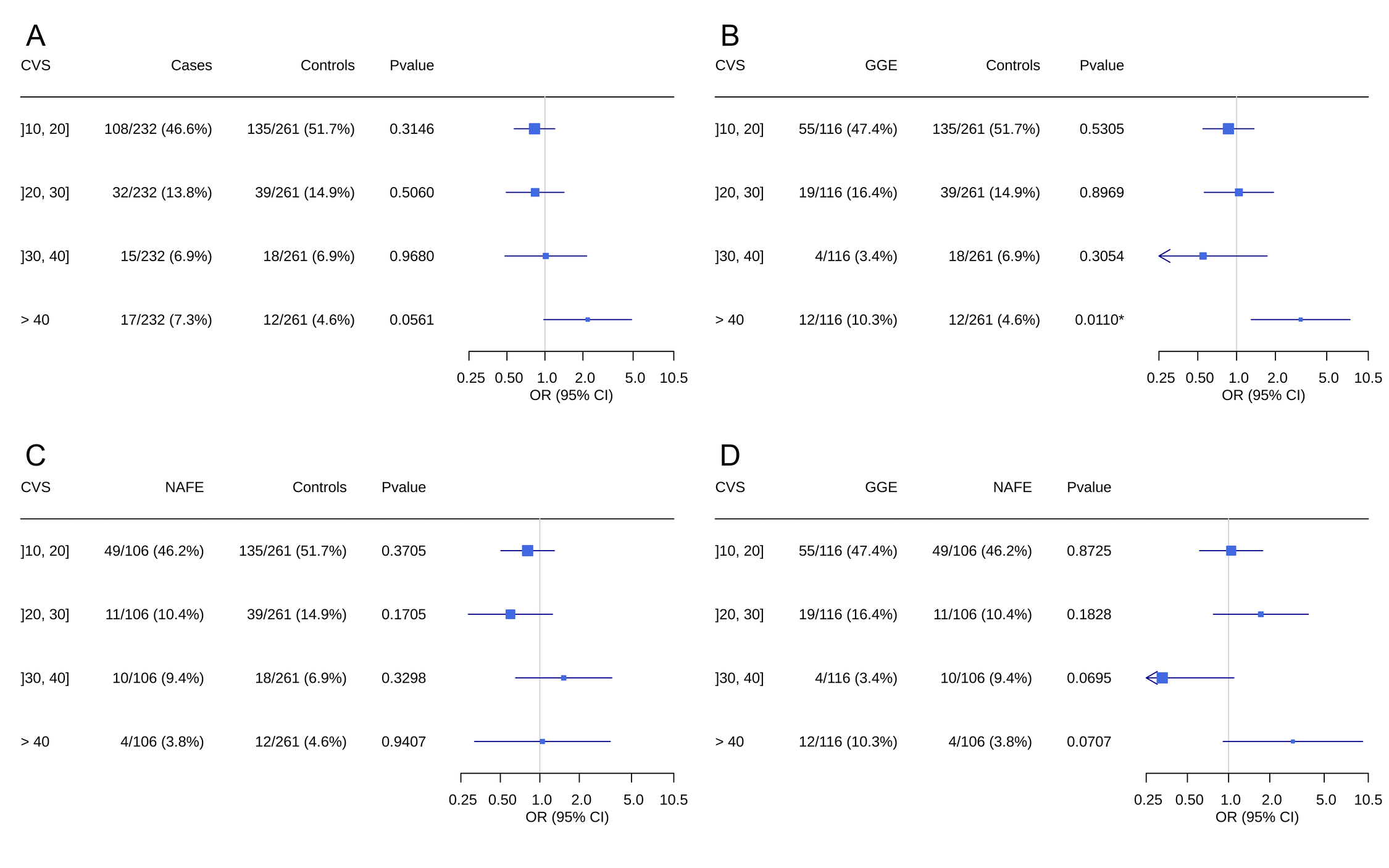

Supplement: S4 Fig — Odds ratios and p-value were calculated using a binomial logistic regression for variants of different Constraint Violation Score (CVS) thresholds. Lines represent 95% confidence intervals. Comparisons were made for cases and controls (A), Genetic Generalized Epilepsy (GGE) and controls (B), Non-Acquired Focal Epilepsy (NAFE) and controls (C) and GGE and NAFE (D). (TIF) [file pone.0291935.s005.tif]
